# Lessons from the “*Urbanorum* spp.” controversy: a supposed parasite and the need for scientific rigor and quality research in Latin America

**DOI:** 10.1590/0074-02760240144

**Published:** 2025-05-02

**Authors:** Diego Fernando Echeverry, Manuel Andrés Sarria, Gloria Inés Palma

**Affiliations:** 1 Universidad del Valle, Facultad de Salud, Departamento de Microbiología, Cali, Colombia; 2 Universidad del Valle, Escuela de Bacteriología y Laboratorio Clínico, Cali, Colombia

**Keywords:** parasitology, diagnostic techniques and procedures, taxonomy, artifacts, steatorrhea, research design

## Abstract

**BACKGROUND:**

Despite insufficient parasitological and clinical evidence, infections attributed to a putative protozoan named “*Urbanorum* spp.” have been associated with gastrointestinal disease and treated with anti-parasitic drugs.

**OBJECTIVES:**

This study aimed to clarify the nature of “*Urbanorum* spp.” and provide guidance for health and biomedical professionals encountering this structure in human stool, emphasising the importance of rigor and quality in biomedical research.

**METHODS:**

Coprological analyses were employed to detect intestinal parasites, lipids, and “*Urbanorum* spp.” in 249 samples. Samples positive for “*Urbanorum* spp.” underwent staining with trichrome, acid-fast, and Sudan IV and contrasted with positive controls. Examination with polarised light microscopy and a fragility test using ethanol were conducted.

**FINDINGS:**

Of the tested samples, 19.4%, 2.5% and 1.3% were positive for intestinal parasites, lipids, and “*Urbanorum* spp.” respectively. Following trichrome and acid-fast staining, few “*Urbanorum* spp.” structures remained intact and exhibited no discernible eukaryotic characteristics; Sudan IV stain, polarized light microscopy and fragility test approaches indicated a cholesterol-based content.

**MAIN CONCLUSIONS:**

“*Urbanorum* spp.” is not a protozoan parasite; therefore, antiparasitic drugs are unwarranted. This structure should be identified as lipid-based material and investigated for possible malabsorption syndrome. Rigorous scientific standards were missed in related publications and peer review, contributing to the spread of this pseudoparasitism case.

Almost a century ago, professor JG Thomson of the Department of Protozoology in the London School of Hygiene and Tropical Medicine wrote: “*In the use of the microscope there is without doubt no more difficult art to be acquired than the ability to interpret all the objects, protean in their character, met with in faecal examination”*.^1^ This statement is still critical for parasitologists, or microbiologists involved in clinical diagnosis. Different artefacts such as undigested plant cells, fibres or fragments, pollen grains, starch granules, air bubbles, lipid droplets, epithelial cells, leucocytes, red blood cells, fungal spores, algae, yeasts, and mite eggs among others, may be erroneously identified as parasites during coprological tests, affecting related medical and therapeutic outcomes for patients^2^ ^,^ ^3^ ^,^ ^4^ ^,^ ^5^ and potentially raising parasitological controversies associated with pseudoparasitism. Parasite diagnosis is a complex process and requires high levels of judgement, interpretation and training, incorporating theoretical education in parasitology alongside visual resources, textbooks, and atlases to support the identification of parasites and distinguishing artefacts.^3^ ^,^ ^4^ ^,^ ^5^

Current available evidence supporting “*Urbanorum* spp.*”* as a new human pathogenic protozoan is debatable from a scientific perspective. A 2013 publication,^6^ states that it was observed for the first time in 1994 in patients from the city of Barrancabermeja (Colombia) and was described morphologically as a hyaline spherical structure with a diameter of up to 100 µm, surrounded by a double-thick membrane with pseudopod-like structures emerging from one or two pores, whose function was assumed related to locomotion or feeding. Based on these features and the suggested asexual reproduction, it was taxonomically classified as an amoeba (Cicloposthidae).^6^ ^,^ ^7^ ^,^ ^8^ This potential protozoan, described only in Latin American countries, was suggested to be transmitted through faecal-oral route and attributed gastrointestinal symptoms and physicochemical alterations of human stools.^6^ ^,^ ^7^ ^,^ ^9^ ^-^ ^13^ Some physicians have provided antiparasitic therapy as treatment with apparent success.^9^ ^,^ ^11^ ^,^ ^12^ ^,^ ^13^ ^,^ ^14^ ^,^ ^15^

Other authors have suggested that “*Urbanorum* spp.*”* could be an artefact, vegetable residues, adipose cells, or fat droplets in human faeces.^16^ ^,^ ^17^ ^,^ ^18^ ^,^ ^19^ ^,^ ^20^ However, the controversy on this supposed protozoan remains fraught with some personnel providing parasitological diagnosis or medical services. The absence of a formal description and the lack of biochemical, ultrastructural (identification of cellular structures), and genetic studies associated with “*Urbanorum* spp.*”*, raise questions about its status as a living organism.^18^ ^,^ ^19^ ^,^ ^20^ ^,^ ^21^ In parasitology, accurate diagnosis requires a combination of classical and modern molecular methodologies.

Classical parasitological approaches include a detailed morphological examination involving size, shape, and recognition of internal structures, while *in vitro* cultures facilitate species identification and increase parasite biomass, which can be used in functional and biological experiments, such as testing Koch’s postulates.^22^ Molecular methods, such as polymerase chain reactions (PCR), sequencing, and other genomic analyses, provide essential genetic data to confirm diagnoses or uncover novel species. However, misidentifications or premature hypotheses about the discovery of new microorganisms can arise, leading to negative implications for both biomedical research and clinical practice. The case of “*Urbanorum* spp.” highlights the critical importance of rigorous validation using multiple lines of evidence before designating it as a new pathogenic species. The purpose of this study was the application of various techniques used in the parasitological diagnostic laboratory and a comprehensive literature review, to demonstrate that “*Urbanorum* spp.” is not a protozoan. This study aimed to clarify the nature of “*Urbanorum* spp.” and provide guidance for health and biomedical professionals encountering this structure in human stool, emphasising the importance of rigor and quality in biomedical research.

## MATERIALS AND METHODS

*Study design and study area* - An observational descriptive study was carried out during five months (November-December 2021 and January-March 2022). Identification of human intestinal parasites was accomplished at the laboratory “Laboratorio de Diagnóstico de Agentes Biológicos - LDAB”, Department of Microbiology, Universidad del Valle, Cali, Colombia, by two expert parasitologists. The faecal specimens used in this study were collected from patients at three public hospitals in Cali during routine medical attention. All positive samples for “*Urbanorum* spp.” were analysed in detail (convenience sampling) by different laboratory techniques (in duplicate) as described below.

*Coprological tests for screening “Urbanorum spp.”* - Faecal human samples were prepared for direct smear examination with 0.85% saline solution and Lugol’s iodine stain. The zinc sulphate flotation concentration protocol was also performed.^23^ ^,^ ^24^ Both methods allowed the detection of parasite cysts, trophozoites, eggs, as well as structures with morphology compatible with “*Urbanorum* spp.” as previously described.^6^ ^,^ ^7^ Thin faecal smears were prepared from positive samples for “*Urbanorum* spp.*”* and processed using different stains and techniques (see below). All samples were kept at -20ºC.

*Stains and laboratory procedures for studying the nature of “Urbanorum spp.”* - Classical parasitological methods, including staining techniques and laboratory procedures were employed to investigate the morphology and content of “*Urbanorum* spp.”:

(i) Trichrome stain: The trichrome stain is used for diagnosis and quality control in specialised parasitology laboratories. The morphology and cellular structures of protozoan parasites can be observed and is more sensitive than wet mount examinations. An aliquot of faecal samples positive for “*Urbanorum* spp.*”* was preserved for a minimum of 24 h in Schaudinn’s fixative solution.^5^ ^,^ ^23^ ^,^ ^24^ After several washes with distilled water using centrifugation, samples were fixed on glass slides using Meyer’s adhesive mixture. The fixed smears were stained with the trichrome dye, acids and alcohols as previously described.^5^ Samples processed with this same protocol and positive for *Balantioides coli* and *Entamoeba histolytica/dispar/moshkovskii* (*Entamoeba* complex) were used as comparators for the recognition of organelles or intracellular structures.

(ii) Acid-fast staining procedure: This technique allows the detection of *Cryptosporidium* spp, *Cystoisospora belli*, and *Cyclospora cayetanensis* oocysts. Both Kinyoun and Ziehl-Neelsen techniques were applied to prepare thin faecal smears positive for “*Urbanorum* spp”.^2^ ^,^ ^5^ For Kinyoun stain, smears were fixed in absolute methanol, stained with carbol-fuchsin, destained in 5% acid-alcohol, and counterstained with methylene blue. In the Ziehl-Neelsen stain, a similar procedure was performed, adding a heating step to increase penetration of carbol-fuchsin through the oocysts wall. For comparison purpose, positive slides processed with the same approaches were available for *C. belli*.

(iii) Sudan IV stain: The Sudan stain based on Sudan IV dye, a lysochrome-diazo dye for staining lipids and lipoproteins on tissues and stools was used. The protocol for Sudan IV stain was standardised for the present study as follows: thin faecal smears (with steatorrhea or “*Urbanorum* spp.”) were fixed in 70% ethanol and stained with Sudan IV dye solution (Sudan IV powder dye, BioGnost Ltd. Zagreb - Croatia, acetone and 70% ethanol), both for 5 min, washed with 70% ethanol and counterstained by progressive hematoxylin for 1 min. Finally, a drop of glycerol was added to the slide and topped with a coverslip. The faecal samples with steatorrhea were used as controls for this technique.

(iv) Polarised light microscopy approach: This laboratory technique uses an optical microscope that is equipped with two 49 mm polarising filters (Izumar PL, Japan). One filter was located between the condenser and the sample (positive for “*Urbanorum* spp.*”* or steatorrhea) and the other between the sample and the observer. This approach allows the identification of the type of fat present in the samples: refringent molecules (anisotropic) are cholesterol esters or free cholesterol, while isotropic molecules such as triglycerides or neutral fats are non-refractive.^25^

(v) “*Urbanorum* spp.” fragility test: Direct evaluation of the fragility of “*Urbanorum* spp.” exposed to 70% ethanol was carried out by mixing aliquots of faecal samples with a 1:1 drop of Lugol’s iodine and 70% ethanol. The homogenised material was covered with a coverslip. The rationale of this approach is to provide additional evidence about the composition of “*Urbanorum* spp.”. If it is composed of lipids, lysis of the structure is expected when exposed to ethanol.

*Microscopic visualisation and image analysis* - The slides were visualised in the Carl Zeiss Axio imager A2 microscope with an AxioCam ERc5s integrated (Carl Zeiss Microscopy, LLC, NY, USA) at 10x - 40x magnifications, while the acid-fast and trichrome stains were at 100x. Photos and morphometric studies were performed with the Zen 3.1 Microscope Software (Carl Zeiss Microscopy, Deutschland GmbH). An Olympus BH2 microscope (Olympus Corporation, Tokyo, Japan) was used for the polarised light approach and photographs were taken with a Motorola G30 cellphone (Motorola Inc, Chicago, Illinois USA).

*Ethics* - Patient consent forms for submitting faecal specimens to the clinical laboratories at the health institutions in Cali include authorisation for storing and using any leftover material for research or teaching purposes. Therefore, this proposal was not reviewed by the research ethics committee at the Universidad del Valle, Cali, Colombia.

## RESULTS

*Coprological examination and analysis* - A total of 249 stool samples from Cali (Colombia) were included for parasitological diagnosis, 237 were successfully processed with the coprological tests and the zinc sulphate concentration. Nineteen point four percent (19,4%) of the samples were positive for intestinal parasites with 37 and eight presenting single and multiple infections respectively. *Endolimax nana* and *Blastocystis* spp. were the most prevalent parasites (Table I). Of the examined samples, 2,5% contained droplets of lipids (steatorrhea) and in 1,3% (three samples) structures compatible with “*Urbanorum* spp.” were observed (two of them with *E. nana* infection) (Table II).

**TABLE I:** Prevalence of intestinal parasites (protozoa and helminths), lipids in stool and “*Urbanorum* spp.” in 237 of 249 stool samples collected in Cali, Colombia. Twelve samples were not included due to insufficient material

Intestinal parasites, lipids, and structures compatible to “*Urbanorum* spp.”	Number of positive samples in 237 processed samples (Percentage)
*Endolimax nana*	14 (5.9)
*Blastocystis* spp.	13 (5,5)
*Entamoeba histolytica/dispar/moskowskii*	11 (4,6)
*Entamoeba coli*	6 (2,5)
*Giardia intestinalis*	4 (1,7)
*Iodamoeba butschlii*	3 (1,3)
*Entamoeba hartmani*	2 (0,8)
*Trichuris trichiura* ^*a*^	1 (0,4)
*Ascaris lumbricoides* ^*a*^	1 (0.4)
*Droplets of lipids (steatorrhea)*	6 (2,5)
*“Urbanorum* spp.*”* ^*b*^	3 (1,3)

*a*: one sample contained both nematodes; *b*: the quotation of “*Urbanorum* spp” across the entire text indicates a figurative meaning.

**TABLE II:** Macroscopic and microscopic description of samples containing the structure named as “*Urbanorum* spp”

Samples	Colour and consistency	Quantity of “*Urbanorum* spp.” structures in 10X per field	Presence of intestinal parasites
1	Brown and formed	3-5	*Endolimax nana* cysts (occasional)
2	Brown and formed	0-1	*Endolimax nana* cysts (occasional)
3	Brown and soft	0-1	Negative

**Fig. 1:**
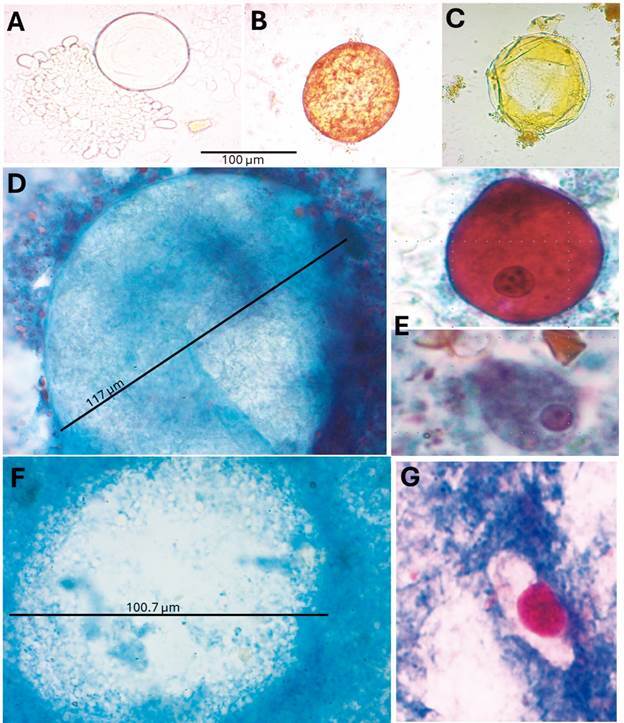
parasitological stains used in the study of the structure “*Urbanorum* spp.” did not indicate it as a living protozoan organism. (A-B) depict typical morphologies of “*Urbanorum* spp.” observed via direct smear examination using saline solution and iodine, respectively (40X magnification); (C) shows a structure in saline solution following the zinc sulphate concentration method (40X); (D) presents an “*Urbanorum* spp.” structure stained with trichrome, revealing absence of eukaryotic structures or organelles, in contrast, (E) displays trophozoites of *Balantioides coli* (top) and *Entamoeba histolytica/dispar/moshkovskii* (bottom) illustrating characteristic features such as cytoplasmic membrane, vacuoles, nucleus, karyosome, locomotion structures among others; (F) depicts a “*Urbanorum* spp.” structure negative for acid-fast stain with undefined borders, while (G) shows an oocyst of *Cystoisospora belli* with a bright-red single sporoblast. D, E, F, and G were visualised at 100X magnification.

In these three positive samples, the structures identified as “*Urbanorum* spp.” were round or slightly oval shaped; size was not consistent, diameters ranged between 80 - 117 µm (Fig. 1A-B). As described by other authors,^6^ ^,^ ^7^ ^,^ ^10^ long, thin, and irregular filamentous projections were visualised in some of them (Fig. 1A). After processing samples with zinc sulphate concentration method, most of the structures were still intact, but some seemed to have lost their content (Fig. 1C). Sample one had a high quantity of “*Urbanorum* spp.” structures as compared to the other samples before the concentration technique (Table II).

*Trichrome and acid-fast stains for studying the morphological characteristics of “Urbanorum spp*.” - Trichrome stain showed that in sample one, three structures with similar size and morphology as “*Urbanorum* spp.” were found in the entire preparation (Fig. 1D) but absent in samples two and three. Well defined membranes, nuclei and organelles were not present in these structures but were clearly identified in the samples of *B. coli,* and the *Entamoeba* complex (Fig. 1E). When the acid-fast stains were analysed, only sample one showed a few structures compatible with “*Urbanorum* spp.”, they were not acid-fast and did not show eukaryotic structures (Fig. 1F). In contrast, the sample with *C. belli* was positive in the preparation (Fig. 1G).

**Fig. 2:**
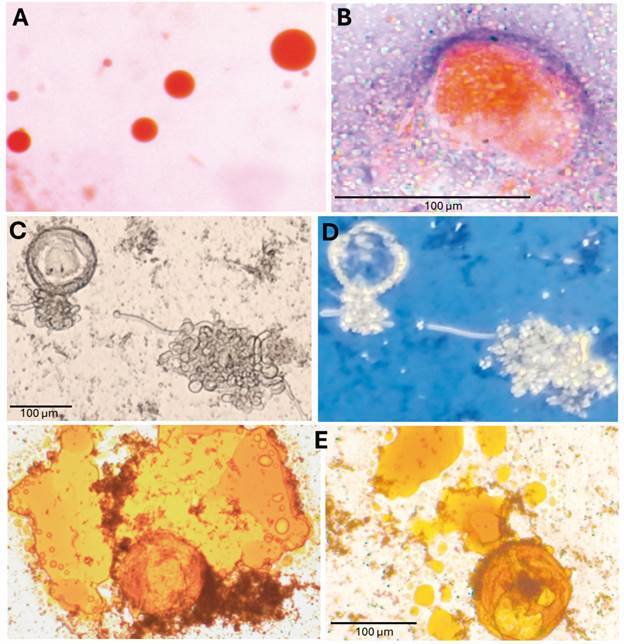
“*Urbanorum* spp.” was identified as a lipid-based structure found in human stools. (A) shows a human stool sample positive for fat due to steatorrhea while an “*Urbanorum* spp.” is shown in (B), both stained with Sudan IV (40X); (C) displays an “*Urbanorum* spp.” with its contents extruded and (D) emphasises the refractive nature of its contents when visualised under polarised light (10X); (E) demonstrates the damage and release of contents from the structure upon direct exposure to ethanol in the presence of iodine (40X).

*Techniques for detecting lipids inside “Urbanorum spp.”* - The Sudan IV stain, polarised light microscopy approach, and ethanol fragility test showed that “*Urbanorum* spp.” is a lipid-based artefact. Stool samples with steatorrhea were included in the study to validate Sudan IV stain. As expected, lipid droplets stained orange (Fig. 2A) as did the “*Urbanorum* spp.” structures, although with less intensity (Fig. 2B). The polarised light microscopy approach showed anisotropic fat inside “*Urbanorum* spp.”, suggesting cholesterol esters or free cholesterol as components (Fig. 2C-D); samples with steatorrhea showed a mixed pattern (isotropic and anisotropic). Finally, the mixed solution of 70% ethanol, aliquot of sample with “*Urbanorum* spp.” and Lugol’s iodine, evidenced the disintegration of the structures contained in the preparation (Fig. 2E).

## DISCUSSION

The original description of “*Urbanorum* spp.” carried out by a parasitology faculty professor in a Colombian university, was published in a general-interest periodical in 2013,^6^ a non-peer-reviewed and not indexed journal (Supplementary data - Table), with no available information regarding the author(s) responsible for the publication. This description was based on: (1) microscopic characterisation using stains such as Lugol’s iodine, modified Ziehl-Neelsen, and methylene blue; (2) functional assays including light sensitivity, cell division, and the analysis of excretion-secretion products; and (3) at least eight epidemiological studies including ~ 15.443 human samples tested for the presence of the “parasite” associated to water consumption.^6^ Although the publication was categorised as a research study by the general-interest periodical, the classical parasitological techniques and functional approaches, as well as the clinical protocols, were not described, and no references were provided.

A review of journals from different databases (PubMed, Scielo, and Google Scholar), written in English, Portuguese and Spanish, excluding grey literature such as undergraduate research, graduate thesis and conference presentations, citing the first description of “*Urbanorum* spp.”,^6^ indicates that humans infected with this presumed parasite may develop watery diarrhoea, dyspepsia, colic, and abdominal pain, with prevalences ranging 0 - 20.8%.^6^ ^-^ ^15^ ^,^ ^26^ ^-^ ^30^ At the same time, stools may show an acid pH, without blood, mucus, or leukocytes.^7^ ^,^ ^9^ ^-^ ^13^ These studies suggested this structure as a new human intestinal pathogen, which may require antiparasitic treatment.

The discovery of a new species, particularly a pathogenic parasite, requires adherence to scientific standards defined by microbiologists and taxonomists. These standards include: (1) an exhaustive review of the existing literature on closely related species;^31^ (2) the use of expert consensus methods for parasite identification;^2^ (3) a framework demonstrating that the organism is a causative agent of disease, contextualised within the evolution of Koch’s postulates;^22^ (4) compliance with the taxonomic principles outlined in the International Code of Zoological Nomenclature;^31^ (5) scientific rigor, which is defined as the application of theoretical or experimental methodologies conducted in a manner that strengthens confidence in the accuracy and reliability of the results^32^ and (6) quality research, associating methodological robustness, valid findings and its contribution of valuable, reproducible, and relevant knowledge to the field.^33^ The initial and subsequent manuscripts related to “*Urbanorum* spp.” (see Supplementary data - Table) fail to meet these criteria. Adherence to these criteria ensures that the identification, characterisation, and classification of a new pathogen are accurate, reproducible, and broadly accepted by the scientific community.

Results of the present study, using detailed classical parasitological approaches, confirmed that the structure named “*Urbanorum* spp.” is not a protozoan in nature. This statement is based on: (1) the inconsistent size of the structures; (2) It did not stain using standard protozoa diagnosis protocols (trichrome and acid-fast stains including human pathogenic parasites as comparators) (Fig. 1D-F); (3) using the trichrome stain, it was possible to demonstrate for the first time, the absence of cytoplasmic membrane, organelles and cytoplasmic inclusions, features of eukaryotic cells (Fig. 1D), key evidence missed in previous publications;^20^ ^,^ ^21^ (4) fat droplets from human samples with steatorrhea as well as *“Urbanorum* spp*.”* stained with Sudan IV, confirming the presence of lipid molecules as previously described^20^ (Fig. 2B); (5) the polarised light approach suggests that the content of the globular structure is cholesterol (Fig. 2D), which is in line with previous findings;^20^ and (6) when exposed directly to alcohol, the structure is destroyed (Fig. 2E). According to this study, the globular structure named “*Urbanorum* spp.”, is a lipid-based structure composed of cholesterol. As expected, by November 2024, neither the International Commission on Zoological Nomenclature (www.iczn.org), nor the International Society of Protistologists (https://protistologists.org/) contain taxonomic records related to “*Urbanorum* spp.” as an official scientific genus, or as a new member in the updated taxonomy of medically important parasites.^34^ In fact, the appending “spp.” is to certain extent, acknowledging that “*Urbanorum*” is a bona fide genus. Therefore, we suggest this be dropped from now on, as proposed in the following paragraphs. Similarly, no related genomic sequences were found at the International Nucleotide Sequence Database Collaboration (INSDC) (https://www.insdc.org) which is in line with a previous publication^18^ and with the findings of the present study.

Cholesterol is absorbed mainly in the proximal jejunum, after it has been processed by the pancreatic cholesterol esterase, reassembled and packaged with triglycerides into chylomicroms.^35^ This molecule in stools is derived from several sources including diet, bile, desquamated epithelial cells and intestinal secretions.^36^ An increase in fat excretion in stools, including cholesterol, is defined as steatorrhea, a clinical feature of fat malabsorption, arising from defective digestion and absorption of fats.^37^ The abundance of cholesterol (and other lipids) in stools may be related to multifactorial causes including: high-fat diets, decreased duodenal pH, lost absorptive intestinal surface area, impaired lipid processing by bile acids, intestinal dysbiosis associated with small intestinal bacterial overgrowth (SIBO), pancreatic exocrine insufficiency, defective chylomicron/lipoprotein secretion, lymphatic system disorders^37^ and gastroenteritis. The biosynthesis of the globular cholesterol structures “*Urbanorum*,” with diameters reaching up to 117 µm, remains to be elucidated. The authors of this publication propose that this phenomenon may be attributed to the accumulation of cholesterol molecules, leading to the formation of aggregates of varying sizes within the intestinal environment; understanding this process and its association with the pathophysiology of malabsorption syndromes and steatorrhea serves as an initial foundation, as disrupted lipid absorption/secretion contributes to the abnormal accumulation of cholesterol within the gastrointestinal tract. A recent study suggested that “*Urbanorum*” structures were vegetable cells (60 - 80 µM containing lipids) from *Persea americana* (avocado),^19^ however, the larger size and the absence of eukaryotic features in “*Urbanorum*” found in the present study dispute these findings.

Different studies performed by researchers in Colombia, Brazil, Ecuador, Peru and Mexico have assumed the pathogenic role of “*Urbanorum*”,^6^ ^-^ ^15^ ^,^ ^26^ ^-^ ^30^ causing confusion for clinical laboratory personnel, microbiologists, physicians, and medical specialists. Some of them approached the Parasitology Unit at Universidad del Valle, Cali (Colombia), asking for advice about diagnosis, laboratory reports, clinical interpretation, and treatment for this supposed “new parasite”. Based on the present results, the term “*Urbanorum*” should be abolished from parasite report forms and any parasitological context when the globular structures containing cholesterol are present. Instead, the presence of these structures should be reported in the coprological analysis as positive for fat (classified as scarce, moderate, or abundant). For the medical personnel, this is suggestive of a fat malabsorption syndrome, correlating with the signs and symptoms described for the supposed “*Urbanorum*” infection.^7^ ^,^ ^9^ ^,^ ^13^ The medical personnel must avoid treatment with any antiparasitic drug including metronidazole, secnidazole, albendazole, or nitazoxanide as previously described.^9^ ^,^ ^11^ ^,^ ^12^ ^,^ ^13^ ^,^ ^14^ ^,^ ^15^ This is worrisome since no therapeutic effect is expected and would lead to adverse effects, selection pressure towards drug tolerance or resistance in intestinal parasites and negative effects on the gut microbiome.^38^ ^,^ ^39^

Based on the previous information, “*Urbanorum*” is a case of pseudoparasitism, where organisms or objects (a lipid-based artefact in this case) are mistakenly identified as parasites within a host.^40^ Its morphology is not similar to any protozoan parasites known to infect humans or animals and does not resemble any artefact previously described.^2^ ^,^ ^4^ This pseudoparasitism case appeared in 2013 and spread throughout Latin America by different publications of regional importance (Supplementary data - Table). A total of 13 journal publications^8^ ^-^ ^15^ ^,^ ^26^ ^-^ ^30^ excluding the first description of *“Urbanorum”* ^6^ and an eBook,^7^ based on observational studies or clinical cases, assumed this structure as a parasite. These publications were linked to: universities (n = 5), medical societies (n = 3), editorials (n = 3), a hospital (n = 1) and a National Health Institute (n = 1); all undergo editorial and peer-review processes, nine were free of charge, in one it was not possible to verify any of the associated indexed databases, the range time for publication was between > 0,5 - 12 months, and four were ranked as Q3. For these publications, one is highly suspicious to be a predatory journal.

These publications led to considerable confusion within the healthcare sector and among researchers, prompting incorrect labelling of this structure as a parasite and the unnecessary application of antiprotozoal treatments. In recent years, there has been growing concern about the dissemination of low-quality research in scientific literature and predatory journals,^41^ ^,^ ^42^ highlighting how manuscripts with insufficient peer review process or questionable methods or data, can gain rapid visibility, influencing negatively clinical diagnosis and decisions. Journals and books reviewers as well as editors, university faculties and research scientists, must be in communication and vigilant to ensure that scientific publications follow rigorous research standards (scientific rigor and quality research),^32^ ^,^ ^33^ and reject publications based on controversial methods or results, and those who are based on questionable publications. Faculties and researchers must generate capacities in critical thinking skills in their students or mentees, encouraging not only rely on the conclusions of studies but also critically evaluate the methodology and data presented, helping to prevent the dissemination of false or misleading information that could negatively affect science, patient care and public health systems, such the case of “*Urbanorum*”.
