# Supplementary material for: Lessons from the “Urbanorum spp.” controversy: a supposed parasite and the need for scientific rigor and quality research in Latin America
Source: Mem Inst Oswaldo Cruz. 2025 May 2;120:e240144. doi: 10.1590/0074-02760240144 (PMC12051917; doi:10.1590/0074-02760240144)
Supplement: Supplementary file 1 [file 1678-8060-mioc-120-e240144-s.pdf]

TABLE

Characteristics of bibliographical resources in which the structure named “*Urbanorum* spp” was considered a pathogenic parasite

Bibliographical resource (Reference)	Associated Organisation	Peer reviewed?[b]	Publication fee	Number of Indexed databases verified[c]	Average time for publication in months	Scopus RANK
Cátedra libre[6]	Universidad Industrial de Santander (Colombia)	NO	NO	0	NA	NO
Parasitologia humana e veterianaria[7] (e-book)	Editora Pasteur (Brazil)[a]	YES	YES (50% less than other editorials)	NA	NA	NO
Revista experiencia en medicina[8]	Hospital regional Lambayeque (Peru)	YES	NO	5	1-2	NO
Acta Elit Salutis[9]	Universidade Estadual do Oeste do Paraná (Brazil)	YES	NO	6	2-3	NO
Revista Peruana de Medicina Experimental y Salud pública[10]	Instituto Nacional de Salud (Peru)	YES	NO	12	3	Q3
American Journal of Case Reports[11,13]	International Scientific Information, Inc (United States)	YES	YES (995 USD)	5	1-2	Q3
Revista Brasileira de Medicina de Família e Comunidade[12]	Sociedade Brasileira de Medicina de Família e comunidade (Brazil)	YES	NO	8	12	NO
Magna Scientia[14]	Unidad Central del Valle del Cauca (University) (Colombia)	YES	NO	4	6	NO
Residencia pediátrica[15]	Sociedade Brasileira de Pediatria (Brazil)	YES	NO	9	NA	NO
Annals of Parasitology[26]	Polish Parasitological Society (Poland)	YES	YES (~ 10,4 USD per page)	4	NA	Q3
Revista de Investigación en Ciencias de la Salud[27]	Universidad Veracruzana (Mexico)	YES	NO	1	6	NO
Revista Uningá[28]	Centro Universitario Ingá (Brazil)	YES	YES (~ 54 USD)	27	6	NO
Horizonte Medico[29]	Universidad de San Martin de Porres (Peru)	YES	NO	14	3	NO
International Journal of Development research[d][30]	Intermedial committee of medical journal editors	YES	YES (fee is provided in the acceptance letter)	0	> 0.5	NO

NA: not available (the information was not accessible on the book or journal’s website). The authors attempted to obtain the information by directly contacting the journal staff via email; however, no response was received. *a*: has two editors in chief and 22 editors, founded by Brazilian university faculties; *b*: as stated by the journal, the process involves the participation of both editor(s) and reviewers; *c*: a manual search of databases was conducted to verify the indexing of the journals. The verified databases associated with the bibliographical resources included: latindex (Sistema Regional de Información en Línea para Revistas Científicas de América Latina, el caribe, España y Portugal), Dialnet (Fundación Dialnet - Universidad la Rioja), Imbiomed (Indice mexicano de revistas biomédicas latinoamericanas), Redalyc (Sistema de Información Científica Redalyc® - Red de Revistas Científicas de Acceso Abierto Diamante), Sumários (Sumarios de Revistas Brasileiras), DIADORIM (Diretório de políticas editoriais das revistas científicas brasileiras), LivRe (Revistas de livre acesso), Web of Science - Clarivate™, Scopus®, PMC - PubMed central, Embase® - NML - MEDLINE, EBSCO, Europe PMC, Scielo (Scientific Electronic Library Online), DOAJ (Directory of Open Access Journals), INDEX COPERNICUS INTERNATIONAL (ICI), SCImago journal rank - SJR, LILACS - Biblioteca virtual em saúde - bvs, ROAD (Directory of Open Access Scholarly Resources), Scite_, REDIB (Red Iberoamericana de Innovación y Conocimiento Científico), Dimensions, Associação Brasileira de Editores Científicos - ABEC® Brasil, ASCI (Asian Science Citation Index), MIAR (Matriz de Información para el Análisis de Revistas), BASE (Bielefeld Academic Search Engine), Slicit, OpenAIRE, MIGUILIM (DIRECTORIO DAS REVISTAS CIENTIFICAS ELECTRONICAS BRASILEIRAS), ACADEMIC RESOURCE INDEX - RESEARCHBIB, INTERNET ARCHIVE SCHOLAR, PERIODICA (Indice de Revistas Latinoamericanas en Ciencias), WikiData, EuroPub, Journals Insights, Sherpa Romeo, EZB (Elektronische Zeitschriftenbibliothek), Sudoc (agence bibliographique de Iénseignement supérieur), Fatcat!, OpenAlex, AURA, ZEITSCHRIFTEN DATENBANK, Periodicos, Oasisbr (Portal brasileiro de publicações e dados científicos em acesso aberto), ISNN (International Standard Serial Number), Signatory of Dora; *d*: this journal is included in an extensive list of predatory journal for 2023 (https://predatoryjournals.org).

CC BY
